# Fluid Replacement Versus Fluid Restriction in COVID-19 Associated Hyponatremia

**DOI:** 10.7759/cureus.9059

**Published:** 2020-07-08

**Authors:** Adeel A Khan, Fateen Ata, Waqar Munir, Zohaib Yousaf

**Affiliations:** 1 Internal Medicine, Hamad Medical Corporation, Doha, QAT; 2 Infectious Disease, Hamad Medical Corporation, Doha, QAT; 3 Clinical Research, Dresden International University, Dresden, DEU

**Keywords:** covid-19, hypovolemia, hyponatremia, inappropriate adh syndrome, sars virus

## Abstract

Hyponatremia is one of the most frequently observed electrolyte abnormalities in coronavirus disease 2019 (COVID-19). Literature describes syndrome of inappropriate anti diuretic hormone (SIADH) as the mechanism of hyponatremia in COVID-19 requiring fluid restriction for management. However, it is important to rule out other etiologies of hyponatremia in such cases keeping in mind the effect of an alternate etiology on patient management and outcome. We present a case of hypovolemic hyponatremia in a patient with COVID-19, which unlike SIADH, required fluid replacement early in the disease course for its correction. A 52-year-old Filipino gentleman presented with a three-week history of diarrhea and symptomatic hyponatremia. There was no history of fever or respiratory symptoms. Physical examination revealed a dehydrated and confused middle-aged gentleman. Labs revealed lymphopenia, thrombocytopenia, and severe hyponatremia (108 mmol/L). Blood cultures and stool workup were negative. Severe acute respiratory syndrome coronavirus 2 (SARS-CoV-2) nasopharyngeal swab was positive. Hyponatremia workup excluded SIADH. The patient had hypovolemic hyponatremia due to gastrointestinal (GI) losses and was managed with saline infusion for correction of hyponatremia with improvement in his clinical status. Hyponatremia in COVID-19 is not only secondary to SIADH but can also be due to other etiologies. Hypovolemic hyponatremia should be distinguished from SIADH as these conditions employ different management strategies, and early diagnosis and management of hypovolemic hyponatremia affects morbidity and mortality.

## Introduction

Hyponatremia has been reported in patients with lower respiratory tract infections and can be hypervolemic, euvolemic, or hypovolemic, with different underlying pathophysiological mechanisms [1]. The severe acute respiratory syndrome coronavirus 2 (SARS-CoV-2) infection has been recently reported to manifest as hyponatremia secondary to syndrome of inappropriate anti diuretic hormone (SIADH ) [2-6]. However, hyponatremia can be due to other etiologies as well. With the prevalence of gastrointestinal (GI) symptoms in coronavirus disease 2019 (COVID-19), there is a possibility of hypovolemic hyponatremia secondary to GI loss [7]. A timely clinical judgment based on etiology guides the management strategy.

## Case presentation

A 52-year-old Filipino gentleman, known hypertensive, presented with a one-day history of confusion, fatigue, and two episodes of vomiting. This presentation was accompanied by generalized abdominal pain and nonbloody diarrhea of three weeks' duration. There was no history of fever or respiratory symptoms. Vital signs were within normal limits. Physical examination revealed a dehydrated middle-aged gentleman, inconsistently oriented to time, place, and person, with no focal neurological deficit. The rest of the physical examination was unremarkable. Initial labs revealed lymphopenia, thrombocytopenia, and severe hyponatremia (108 mmol/L). The patient was managed in the medical ICU, where he received hypertonic saline 100 mL thrice, followed by normal saline infusion of 2 L, with a close serum sodium monitoring.

The initial working diagnosis was of gastroenteritis due to an infectious etiology. Blood and stool workup did not detect any common bacterial, viral, or parasitic pathogen. The SARS-CoV-2 nasopharyngeal swab was sent for polymerase chain reaction (PCR), which came back positive. Apart from fluid replacement, the patient was managed conservatively.

Hyponatremia workup excluded SIADH and was secondary to GI loss (Table 1). Timely determination of the etiology of hyponatremia led to successful but gradual improvement in the symptoms and sodium level. The patient improved and was discharged in a stable condition.

**Table 1:** Relevant lab investigations. SARS-CoV-2 PCR, severe acute respiratory syndrome coronavirus 2 polymerase chain reaction; TSH, thyroid stimulating hormone

Investigation	Result	Normal range
White blood cell count	7.8	4-10 x 10^3/uL
Hemoglobin	17	13-17 g/dL
Hematocrit	43.3	40%-50%
Lymphocyte count	0.5	1-3 x 10^3/uL
Platelets	73	150-400 x 10^3/uL
Creatinine	96	62-106 umol/L
Alanine aminotransferase	81	0-41 U/L
C-Reactive protein	52.8	0-5 mg/L
Procalcitonin	0.22	<0.5 ng/mL
Lactic acid	2.3	0.5-2.2 mmol/L
SARS-CoV-2 PCR	positive	Not applicable
Serum sodium	108	136-145 mmol/L
Serum osmolality	225	275-295 mmol/kg
Urine sodium	36	25-40 mEq/L
Urine osmolality	145	150-1150 mmol/kg
TSH	0.92	0.3-4 mIU/L
Serum cortisol level (AM)	557	133-537 mmol/L

## Discussion

COVID-19 is caused by SARS-CoV-2. The mode of transmission is person to person, primarily via respiratory droplets. The most common symptoms are respiratory, but GI symptoms have also been reported [8].

Hyponatremia is associated with COVID-19 [8-9]. Yousaf et al. described the mechanism of hyponatremia in COVID-19 secondary to SIADH as being multifactorial, including increased interleukin-6 (IL-6) levels stimulating antidiuretic hormone (ADH) release [5]. In the case series, all three patients recovered with fluid restriction. However, it is essential to consider other possible etiologies as a cause of hyponatremia in COVID-19.

Measuring the osmolality of serum and urine is essential in the management of hyponatremia. A normal or high serum osmolality would indicate pseudohyponatremia. Once a low serum osmolality affirms the diagnosis of true hyponatremia, urine osmolality is needed to distinguish between SIADH and other causes. Sodium excretion in urine is a marker of the volume status. However, during initial decision making, clinical judgement of volume status is paramount to prevent any delay in treatment. Volume status would be the deciding factor between fluid conservative and fluid replacement strategy. Erroneous treatment leads to increased morbidity, intensive care admissions, and increased duration of stay [10-11]. Also, inappropriate fluid resuscitation is associated with increased pulmonary complications in COVID-19 [10, 12-13].

Our patient was hypovolemic based on a suggestive history and clinical examination. Hyponatremia workup was sent and fluid replacement was started afterwards awaiting results. The workup showed he was appropriately treated as hypovolemic hyponatremia with IV fluid replacement. Hypovolemic hyponatremia has a higher mortality rate than hyponatremia associated with SIADH [14]. Therefore, caution should be exercised before attributing every hyponatremia in COVID-19 to SIADH. An early clinical judgement should consider the volume status of the COVID-19 patients with hyponatremia to decide between fluid restriction and isotonic fluid replacement.

## Conclusions

Hyponatremia in COVID-19 is not only secondary to SIADH but can also be due to other etiologies. It is critical to establish the cause of hyponatremia early in the disease course to guide initial management. Hypovolemic hyponatremia should be distinguished from SIADH as these conditions employ different management strategies, and the management affects morbidity and mortality.
